# Using Serial and Discrete Digit Naming to Unravel Word Reading Processes

**DOI:** 10.3389/fpsyg.2018.00524

**Published:** 2018-04-13

**Authors:** Angeliki Altani, Athanassios Protopapas, George K. Georgiou

**Affiliations:** ^1^Department of Educational Psychology, Faculty of Education, University of Alberta, Edmonton, AB, Canada; ^2^Department of Special Needs Education, Faculty of Educational Sciences, University of Oslo, Oslo, Norway

**Keywords:** word reading, fluency, RAN, serial naming, discrete naming

## Abstract

During reading acquisition, word recognition is assumed to undergo a developmental shift from slow serial/sublexical processing of letter strings to fast parallel processing of whole word forms. This shift has been proposed to be detected by examining the size of the relationship between serial- and discrete-trial versions of word reading and rapid naming tasks. Specifically, a strong association between serial naming of symbols and single word reading suggests that words are processed serially, whereas a strong association between discrete naming of symbols and single word reading suggests that words are processed in parallel as wholes. In this study, 429 Grade 1, 3, and 5 English-speaking Canadian children were tested on serial and discrete digit naming and word reading. Across grades, single word reading was more strongly associated with discrete naming than with serial naming of digits, indicating that short high-frequency words are processed as whole units early in the development of reading ability in English. In contrast, serial naming was not a unique predictor of single word reading across grades, suggesting that within-word sequential processing was not required for the successful recognition for this set of words. Factor mixture analysis revealed that our participants could be clustered into two classes, namely beginning and more advanced readers. Serial naming uniquely predicted single word reading only among the first class of readers, indicating that novice readers rely on a serial strategy to decode words. Yet, a considerable proportion of Grade 1 students were assigned to the second class, evidently being able to process short high-frequency words as unitized symbols. We consider these findings together with those from previous studies to challenge the hypothesis of a binary distinction between serial/sublexical and parallel/lexical processing in word reading. We argue instead that sequential processing in word reading operates on a continuum, depending on the level of reading proficiency, the degree of orthographic transparency, and word-specific characteristics.

## Introduction

Rapid, automatic word recognition is viewed as a crucial component of fluent reading (LaBerge and Samuels, 1974; Perfetti, 1985; Wolf and Katzir-Cohen, 2001). In their seminal paper, LaBerge and Samuels (1974) highlighted the importance of automaticity in word reading, arguing that after practice and exposure, letters in words are consolidated in memory and, thus, multi-letter patterns become unitized and are perceived as a single unit. Similarly, when describing the phases of reading development, Ehri (2005) essentially equated fluency with “sight word” reading. Reading by sight means that seeing a word automatically activates its pronunciation and meaning in long-term memory in a single step. As such, in skilled reading (or sight-word reading), individual words are recognized as unitized, whole entities. In contrast, reading speed is slower during the initial phases of reading development, or when the number of letters increases (in multisyllabic words). This is thought to be indicative of serial processing for computing the pronunciations of words, in which graphemes are mapped into their corresponding phonemes one after the other (Ans et al., 1998; Coltheart et al., 2001; Ziegler et al., 2003). As beginning readers become more skilled, word naming speed increases even for longer words, consistent with the idea that word reading becomes less serial and more parallel, at least for familiar words (e.g., Di Filippo et al., 2006). Thus, the speed with which a printed word is identified and named has been assumed to reflect the way the stimulus is processed and, subsequently, the level of automaticity in word reading (Bowers and Swanson, 1991). In line with this view, Ehri and Wilce (1983) proposed that we can determine whether familiar words are recognized automatically, as completely unitized symbols, when their naming times have reached the same response rate as the naming of single digits.

In a similar vein, response latency to individually presented digits (or other familiar stimuli presented in isolation) has been used as a measure of the speed of lexical access (e.g., Näslund and Schneider, 1994; Jones et al., 2009; Logan et al., 2011). In fact, the discrete-trial naming task (where individual items are presented in isolation) has been proposed as a much “purer” measure of name retrieval time or item identification speed compared to the serial naming task (where items are presented simultaneously on a grid), because it eliminates more complex processes, such as sequential response, rapid scanning, and motor-production planning involved in the serial format of the task (Stanovich et al., 1983; Wolf, 1991). For example, Bowers and Swanson (1991) examined the relationship between serial and discrete versions of digit naming and a discrete version of reading regular and exception words among Grade 2 readers, and reported that discrete naming was a unique predictor of single word reading. Instead, serial naming did not account for any unique variance in single word reading after discrete naming was controlled. Bowers and Swanson claimed that both digit naming and word reading reflect common lexical retrieval processes.

However, several studies have shown that serial naming is a strong predictor of reading fluency (e.g., van den Bos et al., 2002; Georgiou et al., 2008; Lervåg and Hulme, 2009; Vaessen and Blomert, 2010; Xue et al., 2013; Juul et al., 2014; Moll et al., 2014). In particular, studies that have used both serial and discrete versions of the naming task to predict performance in reading tasks have found that serial naming is a better predictor of reading (e.g., Stanovich et al., 1983; Wagner et al., 1993; Pennington et al., 2001; de Jong, 2008; Georgiou et al., 2013). In light of this evidence, researchers have claimed that serial naming involves processes specific to the sequential nature of the task (e.g., rapid eye movement control and efficient scheduling of multiple items) which drive its relationship with reading (e.g., Georgiou et al., 2013; Gordon and Hoedemaker, 2016; Kuperman et al., 2016), beyond the automaticity of name retrieval (e.g., Stanovich et al., 1983; Logan et al., 2011). Recent studies examining this *serial superiority effect* in the relationship between naming and reading have shown that the way items are presented and processed in both word reading and digit naming can influence the size of their relationship (e.g., de Jong, 2011; Protopapas et al., 2013; Altani et al., 2017b).

In particular, de Jong (2011) argued that measures of serial and discrete naming of digits can provide insight into the processes involved in word reading. Individual word reading (henceforth, *discrete reading*) and discrete naming share the demand of rapid lexical access and retrieval from long-term memory. As such, a strong correlation between the two tasks could be indicative of a sight-word reading process. In contrast, the serial version of the naming task—where items are presented simultaneously in a grid format—taps additional processes specific to its sequential nature. Thus, serial naming reflects serial processing demands beyond its shared processes with discrete naming. That is, serial naming and discrete naming presumably involve the same cognitive processes (e.g., identification of visual information, print-to-sound mapping, articulatory demands) except for the component of sequential processing (e.g., Logan et al., 2011). Hence, it has been argued that a strong relationship between serial naming and reading arrays or lists of words (henceforth, *serial reading*) reflects similar task demands of sequential processing over series of items. By analogy, a strong relationship between serial naming and discrete word reading reflects a serial decoding strategy in word recognition, because in this case, sequential processing concerns series of items (i.e., letters, graphemes, or syllables) *within* individual words.

Evidence from Dutch (de Jong, 2011; van den Boer and de Jong, 2015), a relatively transparent orthography, suggests that the pattern of relationships is in favor of a more serial decoding strategy in the early phases of reading development (Grades 1 and 2), in which stronger correlations between discrete word reading and serial naming were observed. In contrast, in later phases of reading development (Grades 4 and 5), or amongst groups of more advanced readers, discrete word reading is more strongly associated with discrete naming, reflecting sight-word reading. Protopapas et al. (2013) found similar results in Greek, which is also a relatively transparent orthography. More specifically, they reported format-specific relationships between naming and reading tasks among a group of older children (Grade 6), that is, a stronger association between discrete naming and discrete reading, and between serial naming and serial reading. In contrast, a strong association was found between serial naming and both discrete and serial reading among a group of younger children (Grade 2). Thus, although serial naming is strongly associated with serial reading throughout development, its relationship with discrete reading can be indicative of word reading processes when compared to discrete naming across different ages.

Expanding on this rationale, van den Boer and de Jong (2015) proposed that readers can be divided into two groups (or classes), namely *serial* and *parallel* processors, based on the size of the relationship between discrete word reading and serial vs. discrete naming. In their study, they found that when clustering Grades 2, 3, and 5 readers using factor mixture analysis, two classes emerged, representing beginning and more advanced readers. Within the first class of (mostly younger) readers, serial naming correlated more strongly with discrete reading, reflecting serial word processing strategies. In contrast, among the (more advanced) readers in the second class, discrete naming correlated more strongly with discrete reading, reflecting sight-word processing.

Additional findings from a cross-linguistic study have confirmed the strong relationship between discrete naming and discrete word reading among Grade 5 Dutch- and English-speaking children (van den Boer et al., 2016). van den Boer et al. (2016) used both real words and nonwords varying in length and found that discrete naming correlated more highly with discrete reading of short words and nonwords, as well as with longer words, in both languages. This suggests that advanced readers recognize words as whole entities in both relatively transparent and opaque orthographies. Yet a stronger correlation was reported between serial naming and multisyllabic nonword reading in Dutch, whereas in English the relationship of longer nonwords with serial naming and discrete naming was equally strong. van den Boer et al. (2016) argued that long nonwords required a more serial withinword processing strategy in Dutch. In contrast, Grade 5 readers in English had to rely on larger orthographic units to reliably recognize longer nonwords, because individual graphemes are not very reliable units in opaque orthographies (Ziegler and Goswami, 2005).

The studies examining the relationship between serial/discrete naming and serial/discrete reading across ages have so far been conducted in relatively consistent orthographies (Dutch and Greek). Studies in English (an opaque orthography) have examined only advanced readers (van den Boer et al., 2016) or only discrete reading (Bowers and Swanson, 1991). Even though serial naming appears to predict discrete reading in younger readers (Grades 1 and 2) in transparent orthographies, there is some evidence (Bowers and Swanson, 1991) to the effect that discrete naming, reflecting unitized item processing, is more strongly associated with discrete reading among younger readers (Grade 2) in English. It has thus been claimed that a serial decoding strategy is less efficient in opaque orthographies, where grapheme-to-phoneme mappings are not reliable (Ziegler et al., 2010). To address this matter, the present study examined the association between digit naming and word reading, in both serial and discrete formats and over a wide range of grade levels (1, 3, and 5) in English, in order to understand the role of serial and discrete naming as indexes of word reading processes across development in an opaque orthography. To our knowledge, this is the first study to systematically examine the relationship between serial and discrete naming and serial and discrete reading in English, including beginning, intermediate, and advanced readers.

Based on de Jong’s (2011) findings, we would expect the following: (a) Serial naming will strongly correlate with serial reading across grades, reflecting sequential processing across multiple items in both tasks, that is, sequences of digits and sequences of words. (b) The relationship between discrete naming and discrete word reading will increase across grades, reflecting attainment of efficient whole-word processing (i.e., “sight word” reading) rendering words effectively equivalent to unitary symbols. In conjunction with that, (c) the relationship between serial naming and discrete word reading will decrease across grades, reflecting diminishing within-word sequential processing (e.g., serial letter-by-letter or grapheme-by-grapheme) as words are increasingly read “by sight.” Moreover, we would expect serial naming to independently contribute to single word reading, after discrete naming is controlled, only among younger readers, who are expected to still employ sequential within-word processing. In contrast, among older readers, serial naming should not account for additional variance in discrete word reading when discrete naming is controlled, because sight word reading specifically precludes partial, serial processing. However, if readers in English rely on larger units than phonemes to efficiently recognize words from early stages of reading, then discrete naming should be the main predictor of discrete word reading across grade levels.

In addition, we examined whether children from different grades can be grouped into two classes of readers. Based on previous findings in Dutch (de Jong, 2011), most Grade 1 children should be assigned to a “beginner” class of readers, purportedly processing individual words in a serial manner; therefore serial naming should be the main predictor of discrete word reading in this class. In contrast, most Grade 5 children should be assigned to the “advanced” class of readers, purportedly processing words in a parallel manner; therefore discrete naming should be the main predictor of discrete word reading in this class. Finally, because by Grade 3 children have largely mastered word recognition skills (e.g., Kuhn and Stahl, 2003), we expected that the majority of children from this grade level would be grouped together with the more advanced Grade 5 readers.

## Materials and Methods

### Participants

Four hundred twenty-nine English-speaking Canadian children from Grades 1, 3, and 5 (Grade 1: *N* = 167, 87 girls, age *M* = 81.41 months, *SD* = 4.22; Grade 3: *N* = 137, 64 girls, age *M* = 105.75, *SD* = 3.94; Grade 5: *N* = 125, 70 girls, age *M* = 129.70, *SD* = 4.17) participated in the study. All children were recruited on a voluntary basis from eight public elementary schools located in different parts of Edmonton (to represent as much as possible different demographics in our study). The schools can be characterized as average-performing (based on Provincial Achievement Tests) serving primarily middle-class families (based on parents’ education and teachers’ reports). Based on the schools included in our study and the demographics of the students they have traditionally been serving, our sample could be considered representative of the general student population of Alberta. All children were native speakers of English (English language learners who did not have at least 3 years of schooling were excluded to avoid confounding the effects of learning English at the same time as learning to read) and had no formal diagnosis of intellectual, behavioral, or sensory difficulties. Parental and school consent, as well as research ethics approval, were obtained prior to testing.

### Materials

Materials consisted of digits and words. The naming tasks included nine repetitions of four digits (2, 3, 5, and 6). The reading tasks included two sets of 36 high frequency words. All items in the naming and reading tasks were monosyllabic words, varying in length between three to five letters. Also, items were matched between the naming and reading tasks in several variables, including frequency, number of phonemes, number of graphemes, and syllabic structure, in order to keep naming demands constant across naming and reading conditions to the extent possible. Word frequencies were derived from the Children’s Printed Word Database, which includes words that appear in books for children in Grades 1–4 (Masterson et al., 2010).

### Procedure

**FIGURE 1:**
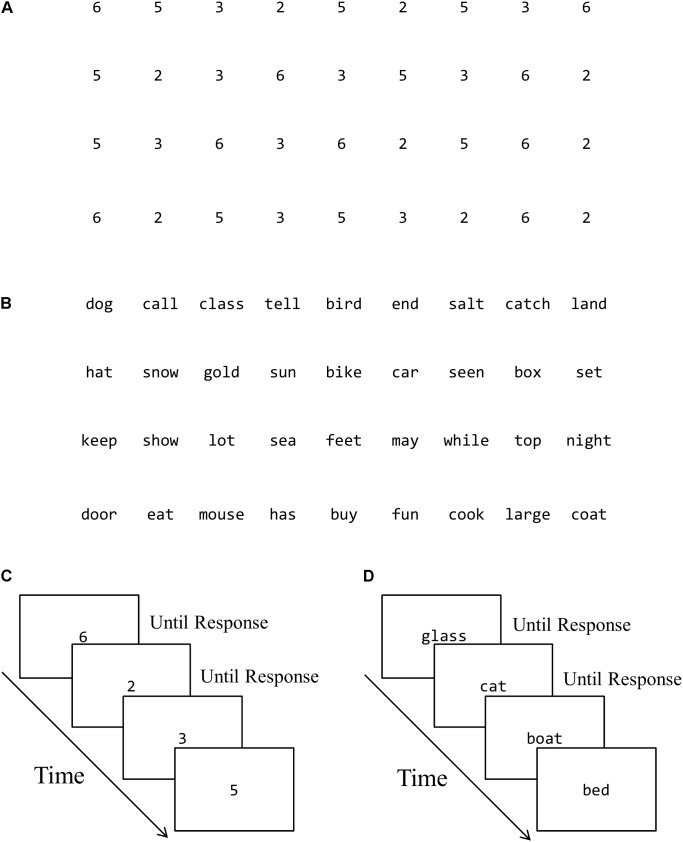
Examples of task presentation and trial sequence. **(A)** Serial digit naming. **(B)** Serial word reading. **(C)** Discrete digit naming. **(D)** Discrete word reading.

Digit naming and word reading tasks were presented in both serial and discrete format. In the serial format, all 36 digits were presented in a matrix of four rows by nine items. All 36 words of the serial reading task were also arranged in a 4 rows × 9 items format to match the presentation of the serial naming task. In the discrete format, all items of the naming and reading tasks were presented one-by-one in the middle of the screen in a fixed quasi random order precluding immediate repetitions (**Figure 1**). For both serial and discrete tasks, children were asked to name out loud the items or read the words as quickly as possible. Instructions and practice items were provided prior to each trial to ensure compliance with task demands.

Item presentation and response recording was controlled by the DMDX experimental display software (Forster and Forster, 2003). Items were presented in black 20-pt Consolas font on a white background and remained on the screen until the experimenter pressed a key to proceed to the next item, as soon as complete production of a response was registered. Individual responses were recorded in audio files through a head-mounted microphone.

Testing took place in April–June (near the end of the academic year). The naming and reading tasks were administered in random order during a 40-min session within a larger testing battery. Children were tested individually in their school during school hours by trained assistants.

## Results

### Data Preparation

Total naming or reading time was determined off-line using CheckVocal (Protopapas, 2007). For serial tasks, total naming or reading times of the entire array were processed; for discrete tasks, naming or reading times of individual items were processed. All recorded response times (RTs) analyzed below included both onset latency and articulation time, to be fully comparable across formats. RTs were subsequently transformed to a scale of “items per second”. For discrete tasks, a single score for each participant was computed by averaging RTs across correctly named or read items. Intraclass correlation coefficient (ICC) for a two-way mixed model was computed to estimate inter-rater reliability (IRR) for a sub-sample of mean response times (across 22 subjects and 2 raters) using icc function from irr package in R (McGraw and Wong, 1996; Gamer et al., 2012). The ICC can range from 0 to 1, with higher ICC (close to 1) indicating smaller-magnitude disagreements (Hallgren, 2012). The resulting ICC was high (0.99; 95%CI: 0.98–0.99), indicating excellent IRR in coding response times and suggesting that a minimum amount of measurement error was introduced in data processing by independent coders.

Errors in serial digit naming were ignored. Errors in serial word reading were analyzed and an accuracy level of 70% correct was used as a cut-off score. This criterion was selected based on previous evidence showing that speed of word recognition begins to develop only when this basic accuracy level of 70% correct is achieved among children in early elementary school grades (Juul et al., 2014). **Table 1** shows the descriptive statistics on each measure excluding data points associated with outliers (three children in Grade 3 and three in Grade 5), accuracy below 70% in either discrete or serial reading tasks (65 children in Grade 1 and one child in Grade 3), overall accuracy <67% (three children in Grade 1), or technical problems (two children in Grade 1 and five children in Grade 3). This cleaning procedure left us with 99 complete cases in Grade 1, 129 in Grade 3, and 122 in Grade 5. Examination of Q–Q plots and Shapiro–Wilk tests indicated no significant deviations from normality. All analyses were conducted using R (R Development Core Team, 2016) with the cleaned-up dataset. Results from previous studies reporting concurrent correlations (or regressions) are included in the Supplementary Material (Supplementary Tables S1–S4, S7) for comparison. Results for the hierarchical regression analyses (Supplementary Table S4) of the sample reported in Protopapas et al. (2018) were derived from a re-analysis of the original dataset.

**Table 1:** Descriptive statistics with the cleaned-up dataset in each grade.

	Grade 1					Grade 3					Grade 5				
	*N*	*M*	*SD*	Skew	Kurt	*N*	*M*	*SD*	Skew	Kurt	*N*	*M*	*SD*	Skew	Kurt
**Serial**														
Digits	99	1.23	0.30	0.12	-0.05	129	1.69	0.39	0.27	-0.20	122	1.92	0.38	0.29	-0.38
Words	97	0.94	0.38	-0.04	-0.93	129	1.57	0.38	-0.14	-0.57	122	1.80	0.37	0.11	-0.40
**Discrete**														
Digits	99	0.85	0.14	0.25	-0.55	129	1.03	0.14	0.04	-0.17	122	1.17	0.13	0.01	0.38
Words	99	0.72	0.16	0.01	0.09	129	0.98	0.13	0.09	0.66	122	1.09	0.12	-0.30	-0.03

*Skew, skewness; Kurt, kurtosis. The scores are presented in items per second (words or digits).*

### Correlation Analyses by Grade

**Table 2** presents the correlation coefficients among discrete and serial versions of digit naming and word reading in each grade. Correlations between serial words and serial digits were moderate to strong (*r* = 0.30–0.61) across the three grades. Correlations between discrete digits and discrete words were strong already by Grade 1 (*r* = 0.51) and remained strong across grades (Grade 3: *r* = 0.64; Grade 5: *r* = 0.78); whereas correlations between serial naming and discrete words remained relatively weaker across grades (Grade 1: *r* = 0.36; Grade 3: *r* = 0.18; Grade 5: *r* = 0.42). The correlation between serial and discrete versions of words or digits was strong in Grade 1 (words: *r* = 0.78; digits: *r* = 0.57), yet moderate in Grade 5 (words: *r* = 0.49; digits: *r* = 0.39). A set of scatterplots among serial and discrete tasks for each grade is also provided in the Supplementary Material (Supplementary Figures S1–S3).

**Table 2:** Correlations (Pearson’s *r*) among discrete and serial digit naming and word reading across grades.

	s_Words		d_Words		s–d correlations	
	d_Digits	s_Digits	d_Digits	s_Digits	Words	Digits
Grade 1	0.24	0.30	0.51	0.36	0.78	0.57
Grade 3	0.26	0.56	0.65	0.18	0.52	0.33
Grade 5	0.34	0.61	0.78	0.42	0.49	0.39

*d, discrete; s, serial.*

### Regression Analyses by Grade

Because serial and discrete naming share several important components (e.g., mapping from print to sound, rapid retrieval of the lexical code, articulatory demands), we performed hierarchical regression analyses to examine the unique contribution of the serial and discrete dimension of the naming task to word reading in each grade. Serial and discrete naming tasks were entered into the regression equation either in the first or in the second step in order to examine their effects on serial and discrete word reading separately, after the variable in the first step was controlled. The unique variance accounted for by each task entered in the second step is reported in **Table 3**.

**Table 3:** *R*^2^ Changes in hierarchical regression analyses using serial and discrete digit naming to predict serial and discrete word reading across grades.

	Grade 1		Grade 3		Grade 5	
Digit naming	Serial words	Discrete words	Serial words	Discrete words	Serial words	Discrete words
(1) Serial	0.08^∗^	0.12^∗∗^	0.33^∗∗^	0.03^∗^	0.37^∗∗^	0.17^∗∗^
(2) Discrete	0.01	0.16^∗∗^	0.02	0.37^∗∗^	0.01	0.45^∗∗^
(1) Discrete	0.05^∗^	0.28^∗∗^	0.10^∗^	0.40^∗∗^	0.11^∗^	0.61^∗∗^
(2) Serial	0.04^∗^	0.01	0.25^∗∗^	0.00	0.27^∗∗^	0.01

*^∗^p < 0.05, ^∗∗^p < 0.0005.*

According to de Jong (2011), serial naming reflecting letter-by-letter serial processing should be the main predictor of word reading (both serial and discrete) in early grades, while discrete naming reflecting sight-word reading should become the dominant predictor of word recognition (i.e., discrete reading) in upper elementary school grades. The results in **Table 3** show that when discrete naming was entered first, serial naming accounted for unique variance (4–27%) in serial word reading across grades, whereas discrete naming did not account for unique variance in serial word reading after the effects of serial naming were controlled for. In contrast, no unique variance in discrete word reading was left to be explained by serial naming after discrete naming was entered first in the regression equation—not even in Grade 1.^1^ When serial naming was entered first, the contribution of discrete naming to discrete word reading remained significant across grades (explaining 16–45% of unique variance). Overall, the contribution of discrete naming to discrete word reading increased across grade levels (from 16% of unique variance in Grade 1, to 37% in Grade 3, and 45% in Grade 5), after partialling out the effects of serial naming (see also Supplementary Table S6 for results from commonality analyses performed across grades).

### Analyses by Performance-Based Groups

Following de Jong’s (2011) suggestion that, because of unequal rates of reading skill development, the division of children into grades might not reflect their true classification as readers, but alternatively a classification based on their performance profiles should be preferred, we performed factor mixture modeling analysis to cluster our sample into groups based on task performance patterns. Factor mixture modeling can be used to distinguish latent classes from unobserved sources of heterogeneity of the sample based on mean performances and interrelations of the observed variables (Cagnone and Viroli, 2012). Four variables were used in the current analysis, including serial and discrete digit naming and serial and discrete word reading. Factor mixture modeling was performed using R package OpenMx 2.0 (see Boker et al., 2018, pp. 86–89). Following de Jong (2011; van den Boer and de Jong, 2015; as clarified by de Jong, personal communication, August 2017), we fit a two-class mixture model to cluster participants into two unobserved latent classes reflecting the hypothesized two groups of readers, namely, readers with different patterns of correlations among naming and reading tasks, reflecting different word processing strategies. Because we were interested in modeling the performance levels and their interrelations among the four individual tasks, rather than their shared variance as captured by latent factors, the model included four dummy latent factors with variance fixed at one and freely estimated mean. Each latent was indicated by a single task with residual variance fixed at zero, mean fixed at the observed value, and freely estimated loading. Class probabilities based on Bayes rule (Estabrook, 2010) were used to classify children into the two groups. (The R OpenMx code for this analysis is listed in the online Supplementary Material; see Supplementary Data Sheet 1).

**Table 4** shows the allocation of children to classes for each grade. The correlations between discrete and serial reading and naming tasks in the final two-class solution are shown in **Table 5**. In both classes, serial word reading correlated more strongly with serial naming (*r* = 0.26 in Class 1 and 0.65 in Class 2) than with discrete naming (–0.08 and 0.39, respectively). The two formats of word reading were more strongly correlated in the first class (0.86) than in the second class of readers (0.53). Discrete word reading correlated more strongly with discrete naming (0.79) than with serial naming (0.44) in Class 2, but not in Class 1 (0.15 and 0.28, respectively). In fact, the correlations between discrete naming and both formats of word reading were not significant in Class 1. Thus, only serial naming correlated significantly with discrete word reading among the first group of readers.

**Table 4:** Number of children in each class.

Subgroup	Total	Grade 1	Grade 3	Grade 5
Class 1	83	70	11	2
Class 2	265	27	118	120

**Table 5:** Correlations (Pearson’s *r*) among discrete and serial digit naming and word reading in each class.

	s_Words		d_Words		s–d correlations	
	d_Digits	s_Digits	d_Digits	s_Digits	Words	Digits
Class 1	-0.08	0.26	0.15	0.28	0.86	0.52
Class 2	0.39	0.65	0.79	0.44	0.53	0.47
*Z*	3.85^∗^	3.99^∗^	7.21^∗^	1.44	5.51^∗^	0.52

*d, discrete; s, serial; Z, z-score value to estimate the size of the difference between the two correlation coefficients. ^∗^Statistically significant z-score values at an alpha level of 0.05.*

Comparisons of correlation coefficients between the two classes via z transformation (Cohen and Cohen, 1983) as implemented in the multilevel package (Bliese, 2016), showed that the serial and discrete versions of word reading were more strongly correlated in the first class than in the second class. Moreover, the relationship between discrete reading and discrete naming was higher in the second class than in the first class of readers, whereas the relationship between discrete reading and serial naming did not differ significantly between the two classes of readers.

Finally, hierarchical regression analysis was performed using serial and discrete naming to predict serial reading and discrete reading in each class separately (**Table 6**). In the first class, serial naming was the main predictor of both formats of word reading. In the second class of readers a different pattern emerged, with serial naming being the main predictor of serial reading and discrete naming the main predictor of discrete reading. In fact, the contribution of discrete naming to discrete word reading was significant only in Class 2. In contrast, serial naming was consistently a unique predictor of serial word reading across classes. Notably, the coefficient of discrete naming predicting serial word reading was negative in the first class of readers, while serial naming not only was a unique predictor of serial reading, but also its contribution increased with discrete naming controlled for, signaling the presence of a suppressive effect (see Logan et al., 2011).

**Table 6:** *R^2^* Changes in hierarchical regression analyses using serial and discrete digit naming to predict serial and discrete word reading in each class.

	Class 1		Class 2	
Digit naming	Serial words	Discrete words	Serial words	Discrete words
(1) Serial	0.06^∗^	0.07^∗^	0.42^∗∗^	0.19^∗∗^
(2) Discrete	0.06^∗^	0.00	0.01	0.44^∗∗^
(1) Discrete	-0.01	0.01	0.15^∗∗^	0.63^∗∗^
(2) Serial	0.12^∗^	0.06^∗^	0.28^∗∗^	0.00

*^∗^p < 0.05, ^∗∗^p < 0.0005.*

## Discussion

The purpose of this study was to examine the relationship between serial and discrete naming and reading across three separate grade levels in English, using naming tasks to index word reading processes. Our results showed that (a) the contribution of serial and discrete naming to word reading is distinct beyond any shared variance, and (b) the serial and discrete naming and—especially—reading tasks start off as rather similar, yet their relationship gradually decreases with age and reading proficiency.

### Serial vs. Discrete Dimension in Naming and Reading

One of our main hypotheses was that the relationship between discrete reading and discrete naming should increase with age, as word recognition becomes more automatic and words are perceived as whole units (“sight words”). In contrast, the relationship between discrete reading and serial naming, reflecting serial processing within the word (letter-by-letter), should decrease with age. In line with our hypotheses, the correlation between discrete words and discrete naming increased across grades, consistent with the idea that individual words are recognized as whole units—similarly to single digits—among more advanced readers. In contrast, the relationship between serial naming and discrete reading remained rather stable across grade levels, a finding which is at odds with the idea that serial naming indexes serial within-word processing. This could mean either that Grade 5 readers of English are not sight-word readers, even of short, familiar, and frequent words; or that a correlation with serial naming does not in fact imply within-word serial processing (hence precluding sight-word reading). But is this finding truly novel and discrepant? In fact, a similar pattern of associations between serial naming and discrete reading has been observed in previous studies in transparent orthographies (see Supplementary Table S1), suggesting that the link between the two domains may not strictly reflect a serial item-by-item processing *per se*. Additionally, our results showed that the correlation between serial naming and serial reading increases with age; a finding consistent with previous evidence in other languages (see Supplementary Table S2), suggesting that reading series of words gradually becomes more similar to naming series of overlearned symbols.

To address this conundrum, we examined the unique contribution of serial vs. discrete naming to word reading beyond any shared variance. Serial naming was found to be a unique predictor of serial reading across grades, consistent with previous findings in transparent orthographies indicating a serial superiority effect (see Supplementary Table S4). Notably, serial naming did not account for any unique variance in discrete word reading in our study, not even among younger readers. This finding contradicts previous studies in transparent orthographies (see Supplementary Table S4), in which serial naming was a unique predictor of discrete word reading among novice readers (de Jong, 2011; Protopapas et al., 2018). However, a closer look at the results of the re-analyzed data from Protopapas et al. (2018) suggests that serial naming is not a better predictor of discrete reading compared to discrete naming among Grade 1 Greek readers (see Supplementary Table S4). Similarly, a previous study among Grade 2 English-speaking children found that serial digit naming did not contribute additional variance to individual word reading speed after discrete digit naming was controlled for (Bowers and Swanson, 1991), consistent with our findings.

One could interpret this pattern of results according to theories suggesting that readers in opaque orthographies like English rely mostly on larger orthographic units for efficient word recognition (e.g., Ziegler and Goswami, 2005). Indeed, the absence of a significant effect from serial naming to discrete word reading as early as Grade 1 in our study presumably reflects an increased requirement to proceed faster to unitization of items (i.e., chunking) for efficient word recognition in English. Moreover, the words in our study were chosen to be familiar to the children, and were short and easy enough to be read correctly by most first graders. Therefore, they were especially likely to have attained sight-word status among the better readers in our sample. However, the fact that similar patterns of results were evident in a relatively transparent orthography (Greek) with longer (two-syllable) words (see Supplementary Table S4) suggests that differences based on orthographic transparency cannot entirely explain the observed pattern of results in our study.

### From Binary Distinctions to Flexibly Adjustable Processing

Alternatively, a significant effect from serial naming to discrete reading may imply a more dynamic skill of processing multiple elements in a sequential manner. That is, the extent of sequential processing demands in discrete word reading – reflected by the size of its relationship with serial naming – may vary as a function of word length (within a language) or language-specific characteristics (across languages). In accord with this idea, van den Boer et al. (2016) found that serial digit naming uniquely predicted discrete reading of multisyllabic nonwords among Grade 5 Dutch-speaking children, whereas both serial and discrete digit naming predicted discrete reading of multisyllabic nonwords among Grade 5 English-speaking children, suggesting that within-word sequential processing requirements are affected by item-specific and general orthographic factors, and are therefore not entirely determined by individual differences in general reading skill.

In our study, high-frequency, short and highly familiar words were used, probably limiting the extent to which sequential processing was required for word recognition, even for less advanced readers. This might also explain previous findings in van den Boer and de Jong (2015) showing that serial naming was a better predictor of single word reading compared to discrete naming among Grade 2 readers (see Supplementary Table S4): Their use of words varying in length and frequency may have increased the demand for sequential within-word processing among the younger children they studied. Similarly, evidence from Greek (see Supplementary Table S4; Protopapas et al., 2018) showing that serial naming predicts discrete word reading among the younger Grades 1 and 3 readers might be due to the fact that two-syllable words were used in that study, presumably resulting in at least some sequential within-word processing requirements (see also Altani et al., 2017a, for related discussion).

Thus, the magnitude of the relationship between serial naming and discrete word reading might be dynamically adjusted based not only on grade level or reading proficiency, but also on word-specific or orthographic system characteristics, indicating a sequential processing continuum rather than a binary distinction (i.e., serial vs. parallel processing) either among readers or among items. Notably, the binary theoretical distinction between a supposedly parallel lexical vs. a supposedly serial sublexical reading route has also been challenged by previous studies using serial and discrete naming to index word processes. Specifically, van den Boer and de Jong (2015) and van den Boer et al. (2016) found that naming single digits predicts discrete reading of not only short words but also nonwords among advanced readers, suggesting that reading processes for familiar words and nonwords are similar, in sharp distinction to fundamental dual-route assumptions.

Finally, the finding that discrete naming accounted for unique variance in discrete word reading after serial naming was entered in the regression equation across grades – a finding also observed in other studies (see Supplementary Table S4) – indicates that what is shared between discrete naming and discrete word reading is not included in the shared variance between discrete and serial naming. This may seem contradictory to the notion that serial naming shares everything with discrete naming except for the demands involved in sequential processing, and that, therefore, when accounting for serial naming, no additional variance should be left in discrete reading to be explained by discrete naming. This apparent contradiction can be explained by considering that it is possible for two tasks depending on mostly overlapping processes to be only weakly correlated, if the variance in the non-shared element(s) dominates overall performance. In particular, serial naming seems to be dominated by the ability to sequentially process multiple items, rather than by single element naming processes (evidenced by the moderate correlations between discrete and serial naming). As such, individual differences in serial naming, to a large extent, reflect variability in skills associated with efficient scheduling of sequences, where multiple processes occur simultaneously, rather than with the total recognition time for each individual item within the series. Consistent with this explanation, it has been shown in both our results (see **Table 6**) and previous studies that the effect of serial naming to serial reading increases when discrete naming is entered in the regression equation, indicating suppression from discrete naming to serial reading (e.g., Logan et al., 2011; Protopapas et al., 2013; Logan and Schatschneider, 2014), and thus suggesting that the serial dimension in naming (and reading) is largely independent from the speed with which individual items are processed within the discrete dimension of the tasks.

### Grouping Children Into Classes

As hypothesized by de Jong (2011) and confirmed by van den Boer and de Jong (2015) for Dutch children, our latent class analysis showed that the assumption of equal development among all children within a grade group was not optimal, as about 30% of the children in Grade 1 were assigned to the second class of more advanced readers, along with most of Grades 3 and 5 children, indicating that there is no substantial difference in the way serial and discrete reading and naming tasks are performed between these two grades, at least as can be determined by their patterns of performance and interrelations.

Interestingly, when one third of Grade 1 students were classified as Class 2 readers, the pattern of results for Class 1 (**Tables 5**, **6**) departed from that for the whole of Grade 1 (**Tables 2**, **3**). Specifically, the correlation between discrete naming and discrete reading became insignificant, while serial naming became a significant predictor of single word reading among this group of beginning readers. These findings are consistent with those of a previous study from an orthographically transparent language (Dutch), indicating that the class of novice readers process single words in a serial manner (de Jong, 2011; see Supplementary Table S7). However, a more recent study in Dutch found that discrete reading correlated equally with discrete and serial naming among Class 1 readers (van den Boer and de Jong, 2015; see Supplementary Table S7). These inconsistent findings could be explained by differences in the composition of the first class of readers by students from various grade levels. More specifically, in both our study and de Jong (2011), most of the children assigned to Class 1 were from Grade 1 (see Supplementary Table S8). Instead, in van den Boer and de Jong (2015) most of the children assigned to Class 1 were from Grade 2 (because Grade 1 children were not included in that study; see Supplementary Table S8). At the same time, a large proportion of Grade 2 children in the previous studies was classified into the second group of more advanced readers, where discrete naming was the main predictor of single word reading. Hence, it seems that by Grade 2 (and Grade 3), the majority of children are able to read short familiar words by sight.

In sum, this line of evidence suggests that the degree of sequential within-word processing in the first group of readers is dictated, at least partially, by the level of reading proficiency of the students assigned in the group. At the same time, previous evidence showing that not only serial but also discrete naming correlates significantly with single word reading among the first class of readers, can be indicative of an intermediate phase where readers may process (at least some) words in chunks larger than individual letters, without having fully mastered sight-word reading, thus suggesting that serial and parallel processing of words are not mutually exclusive. In addition, our finding that one third of Grade 1 students were assigned to the second class of readers indicates that a significant proportion of children from this grade level have already mastered sight-word reading, at least for high-frequency, short words.

One clear distinction that emerged between the two classes of readers was in terms of the size of the relationship between serial and discrete word reading. This correlation was very strong in the first class of readers but substantially weaker in the second class (**Table 5**), suggesting that for beginner readers, serial and discrete versions of word reading are almost identical, whereas for the second class of readers, word reading tasks become fairly different depending on their presentation format (serial vs. discrete). This finding is consistent with previous evidence showing a common underlying structure based on task *content* (reading vs. naming) early in development, whereas in later development a task *format* structure (serial vs. discrete) predominates (Protopapas et al., 2013). Thus, other skills associated with sequential processing of multiple items appear to be crucial for emerging serial word reading fluency (Zoccolotti et al., 2015; Gordon and Hoedemaker, 2016; Protopapas et al., 2018), beyond individual item properties or word name retrieval speed. Recent evidence is in line with this idea, suggesting that tasks in which individuals are asked to process strings of visual symbols, requiring rapid eye movement control and efficient simultaneous processing of multiple items, are strong predictors of early and later reading performance (Kuperman et al., 2016; Onochie-Quintanilla et al., 2017).

## Limitations and Conclusion

Some limitations of the present study are worth mentioning. First, we included only high frequency short words and we do not know if similar results would have been observed with multisyllabic words or pseudowords. It is possible —in fact, likely, according to our interpretation—that serial naming would be a stronger, and possibly unique, predictor of discrete reading of longer words or pseudowords, at least during the initial phases of reading development in English. Second, we have not examined the role of other potentially relevant components of individual skill development, such as vocabulary, phonemic awareness, and morphological awareness, which may contribute to individual word reading efficiency (e.g., Hudson et al., 2012; Kim, 2015; Desrochers et al., 2017).

In conclusion, our results demonstrated that both serial naming and discrete naming reflect distinct skills important for word reading efficiency beyond any shared variance. We also found strong correlations between discrete word reading and discrete naming, already present in early development. This is consistent with the psycholinguistic grain size theory (Ziegler and Goswami, 2005), suggesting that children who learn to read in opaque orthographies, like English, may use larger units of information (e.g., rimes) to efficiently recognize words. However, word-specific characteristics, along with orthographic transparency and reading proficiency, may influence the extent to which sequential processing is required within words.

Although our results support the classification of readers into two groups based on whether sequential processing takes place also within words or only between words, our study goes beyond the previous literature by highlighting evidence through comparisons of current and previous datasets that challenge the hypothesis of a binary distinction between serial and parallel word processing. We propose that, instead of a dichotomy between two mutually exclusive opposites (i.e., serial/sublexical vs. parallel/lexical), the temporal sequencing of multi-element processing may occur on a continuum (from simultaneous to consecutive) across items differing in properties such as familiarity, length, and lexicality. This speculative hypothesis should be further examined, including stimuli differing in their psycholinguistic properties and samples from different grade levels and orthographies.

Finally, the distinction between the serial and discrete formats of both naming and reading that emerged in both groups of readers was interpreted as an indication that performance in serial naming (and serial reading, in more advanced readers) relies on additional skills associated with efficient processing and coordination of series of items, beyond the ability to process individual items efficiently. This further implies that processing arrays of simple symbols or unconnected words might reflect important skills, beyond name retrieval speed or knowledge of individual words. Thus, serial naming and its unique role in word reading can be used as an index of emerging sequential processing skills, which may be critical for word fluency development.

## Ethics Statement

This study was carried out in accordance with the recommendations of the University of Alberta Human Research Ethics Board with written informed consent from all subjects (parental consent) in accordance with the Declaration of Helsinki. The protocol was approved by the University of Alberta Human Research Ethics Board and the Edmonton Public School Board.

## Author Contributions

AA conceptualized the research project, developed the measures, collected the data in schools, ran the analyses, interpreted the findings, and took the lead on writing the manuscript. AP contributed to the conception and design of the work, the data analysis and interpretation of the findings, and revised the work critically. GG supervised the data collection, contributed to the conception and design of the work, and supported the writing of the manuscript.

## Conflict of Interest Statement

The authors declare that the research was conducted in the absence of any commercial or financial relationships that could be construed as a potential conflict of interest.

## Footnotes

1 Hierarchical regression analyses including the entire Grade 1 sample (excluding only those students who scored below 30% correct in the serial and discrete word reading) showed that the pattern of results did not differ between Grade 1 children who scored above 70% correct vs. Grade 1 children who scored above 30% correct in both serial and discrete reading tasks (see Supplementary Table S5).
