# Supplementary material for: Using Serial and Discrete Digit Naming to Unravel Word Reading Processes
Source: Front Psychol. 2018 Apr 13;9:524. doi: 10.3389/fpsyg.2018.00524 (PMC5908969; doi:10.3389/fpsyg.2018.00524)
Supplement: Supplementary file 1 [file Table_1.pdf]

Supplementary Material

Using Serial and Discrete Digit Naming to Unravel Word Reading Processes

Angeliki Altani[1*], Athanassios Protopapas[2], George K. Georgiou[1]

[1]Department of Educational Psychology, University of Alberta, Edmonton, AB, Canada

[2]Department of Special Needs Education, University of Oslo, Oslo, Norway

*** Correspondence:** Angeliki Altani: altani@ualberta.ca

1 Supplementary Figures and Tables

1.1 Supplementary Tables

Supplementary Tables S1–S4 and S7–S8 report the same analyses as in the main text together with results from previous studies for comparison. Tables S5 and S6 report complementary analyses to the results of the main text.

Table S1. Correlations (Pearson's *r*) among discrete words with discrete and serial digits across grades for each study

	Grade	Our study	de Jong (2011)	van den Boer & de Jong (2015)	van den Boer et al. (2016)	Protopapas et al. (2013)	Protopapas et al. (2018)
Discrete words-Discrete digits	1	.50	.28	–	–	–	.54
	2	–	.58	.47	–	.50	–
	3	.65	–	.50	–	–	.82
	4	–	.51	–	–	–	–
	5	.78	–	.64	.86	–	.78
	5b	–	–	–	.80	–	–
Discrete words-Serial digits	1	.36	.36	–	–	–	.47
	2	–	.29	.53	–	.42	–
	3	.18	–	.27	–	–	.60
	4	–	.33	–	–	–	–
	5	.42	–	.23	.46	–	.45
	5b	–	–	–	.55	–	–

Note. The upper part of the table displays the correlations between discrete word reading and discrete digit naming. The bottom part of the table displays the correlations between discrete word reading and serial digit naming. 5b is derived from van den Boer et al. (2016) who reported the correlation coefficients for both English-speaking and Dutch-speaking Grade 5 children (5b = correlation coefficients for Dutch-speaking children).

Table S2. Correlations (Pearson's *r*) among serial words with discrete and serial digits across grades for each study

	Grade	Our study	de Jong (2011)	van den Boer et al. (2016)	Protopapas et al. (2013)	Protopapas et al. (2018)
Serial words-Discrete digits	1	.24	.19	–	–	.32
	2	–	.30	–	.26	–
	3	.32	–	–	–	.48
	4	–	.09	–	–	–
	5	.34	–	.42	–	.42
	5b	–	–	.48	–	–
Serial words-Serial digits	1	.30	.34	–	–	.53
	2	–	.50	–	.61	–
	3	.58	–	–	–	.62
	4	–	.53	–	–	–
	5	.61	–	.70	–	.66
	5b	–	–	.80	–	–

Note. The upper part of the table displays the correlations between serial word reading and discrete digit naming. The bottom part of the table displays the correlations between serial word reading and serial digit naming. 5b is derived from van den Boer et al. (2016) who reported the correlation coefficients for both English-speaking and Dutch-speaking Grade 5 children (5b = correlation coefficients for Dutch-speaking children).

Table S3. Correlations (Pearson's *r*) among serial and discrete versions of words and digits across grades for each study

	Discrete digits – Serial digits			Discrete words - Serial words		
Grade	Our study	van den Boer et al. (2016)	Protopapas et al. (2018)	Our study	van den Boer et al. (2016)	Protopapas et al. (2018)
1	.57	–	.40	.78	–	.85
2	–	–	.38 [a]	–	–	.83 [a]
3	.33	–	.49	.52	–	.69
4	–	–	–	–	–	–
5	.39	.40	.42	.49	.53	.56
5b	–	.53	–	–	.57	–

Note. [a] Data from Protopapas et al. (2013); 5b is derived from van den Boer et al. (2016) who reported the correlation coefficients for both English-speaking and Dutch-speaking Grade 5 children (5b = correlation coefficients for Dutch-speaking children).

Table S4. R^2 changes in hierarchical regression analyses using serial and discrete digit naming to predict discrete word reading for each study

	Our Study			Protopapas et al. (2018)			van den Boer & de Jong (2015)		
Digit Naming	Grade 1	Grade 3	Grade 5	Grade 1	Grade 3	Grade 5	Grade 2	Grade 3	Grade 5
1. Serial	$.12^{**}$	$.03^{*}$	$.17^{**}$	$.21^{**}$	$.33^{**}$	$.20^{**}$	$.28^{**}$	$.08^{*}$	$.04^{*}$
2. Discrete	$.16^{**}$	$.37^{**}$	$.45^{**}$	$.15^{**}$	$.37^{**}$	$.41^{**}$	$.06^{**}$	$.18^{**}$	$.39^{**}$
1. Discrete	$.28^{**}$	$.40^{**}$	$.61^{**}$	$.28^{**}$	$.65^{**}$	$.59^{**}$	$.22^{**}$	$.26^{**}$	$.42^{**}$
2. Serial	.01	.00	.01	$.07^{*}$	$.06^{*}$	.02	$.13^{**}$	.00	.01

Note. Results reported for Protopapas et al. (2018) were derived from a re-analysis of the original dataset. * $p < .05$; ** $p < .0005$;

Table S5. R^2 changes in hierarchical regression analyses using serial and discrete digit naming to predict serial and discrete word reading for Grade 1 children scoring above 70% correct versus above 30% correct

	Grade 1 Acc > 70% *N* = 99		Grade 1 Acc > 30% *N* = 144	
Digit Naming	Serial Words	Discrete words	Serial Words	Discrete words
1. Serial	.09**	.12**	.08**	.11**
2. Discrete	.01	.16**	.01	.08**
1. Discrete	.01	.28**	.04*	.17**
2. Serial	.04*	.01	.05*	.02

Note. Acc = Accuracy; The second group (Acc > 30%) includes all Grade 1 children from our study who scored at least 30% correct in both serial and discrete word reading tasks, including those children from the first group (Acc > 70%) who scored above 70% correct on the same reading tasks. * $p < .05$; ** $p < .0005$

Table S6. Variance proportions predicting serial word reading and discrete word reading in each grade

Variable		Serial Words			Discrete Words		
		Unique	Common	Total	Unique	Common	Total
Grade 1							
Serial	Digits	.04	.05	.09	.01	.12	.13
Discrete	Digits	.01	.05	.06	.14	.12	.26
Grade 3							
Serial	Digits	.25	.09	.33	.00	.03	.03
Discrete	Digits	.02	.09	.10	.39	.03	.42
Grade 5							
Serial	Digits	.27	.10	.38	.01	.16	.17
Discrete	Digits	.01	.10	.12	.45	.16	.61

Table S7. Correlations (Pearson's *r*) among discrete and serial versions of digit naming and word reading in each class and each study

		Serial words			Discrete words		
Variable	Class	Our study	de Jong (2011)	van den Boer & de Jong (2015)	Our study	de Jong (2011)	van den Boer & de Jong (2015)
Discrete digits	1	–.08	.06	–	.15	.16	.46
	2	.39	.17	–	.79	.56	.67
Serial digits	1	.26	.60	–	.28	.45	.55
	2	.65	.57	–	.44	.44	.44

Table S8. Number of children per grade in each class and each study

	Grade	Our study	de Jong (2011)	van den Boer & de Jong (2015)
	1	70	50	–
	2	–	20	34
Class 1	3	11	–	2
	4	–	4	–
	5	2	–	1
	1	27	21	–
	2	–	54	83
Class 2	3	118	–	84
	4	–	123	–
	5	120	–	110

1.2 Supplementary Figures

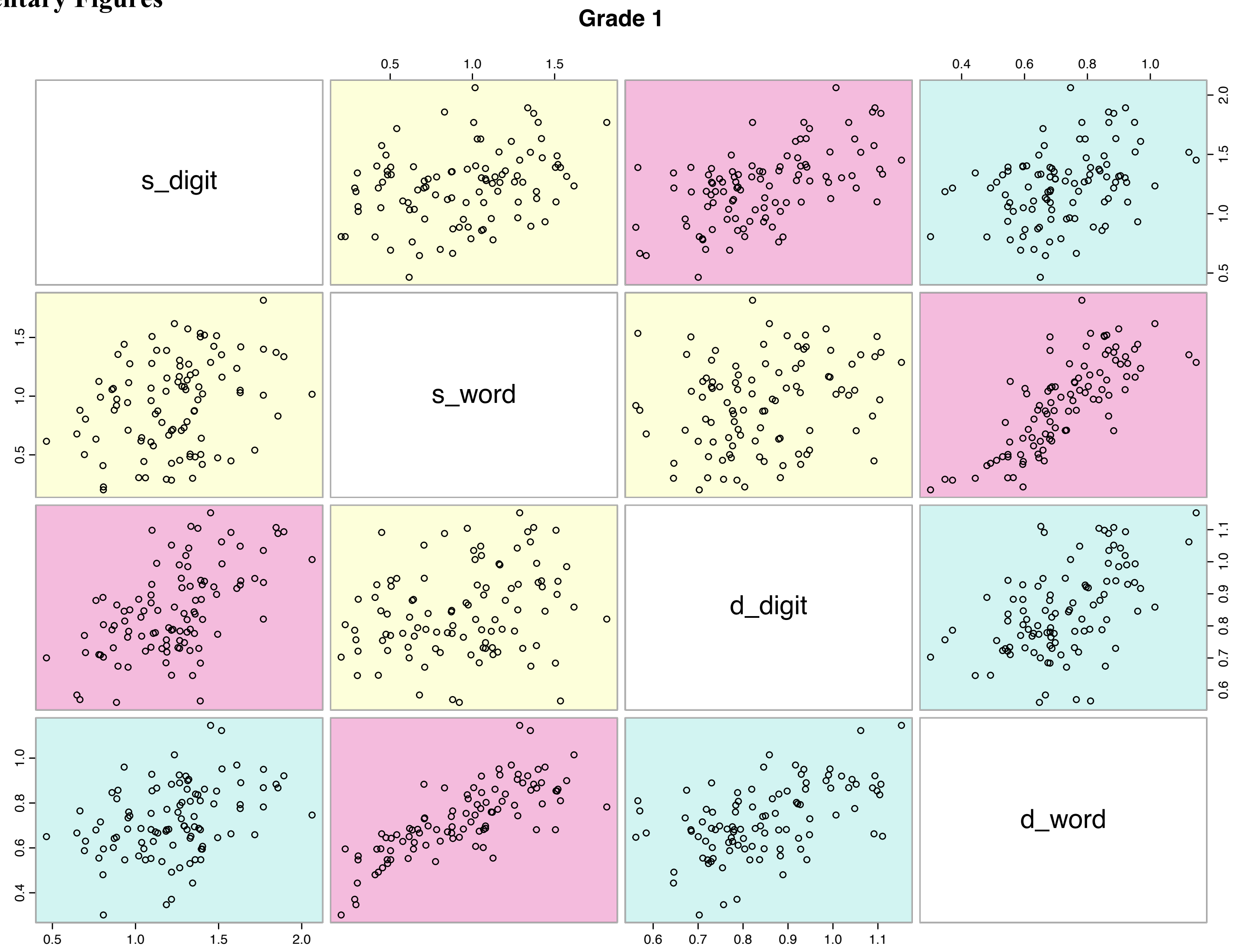

Supplementary Figure 1. Bivariate scatterplots among serial and discrete digits and words in Grade 1. Darker colors indicate stronger correlations. (All data points in items per second).

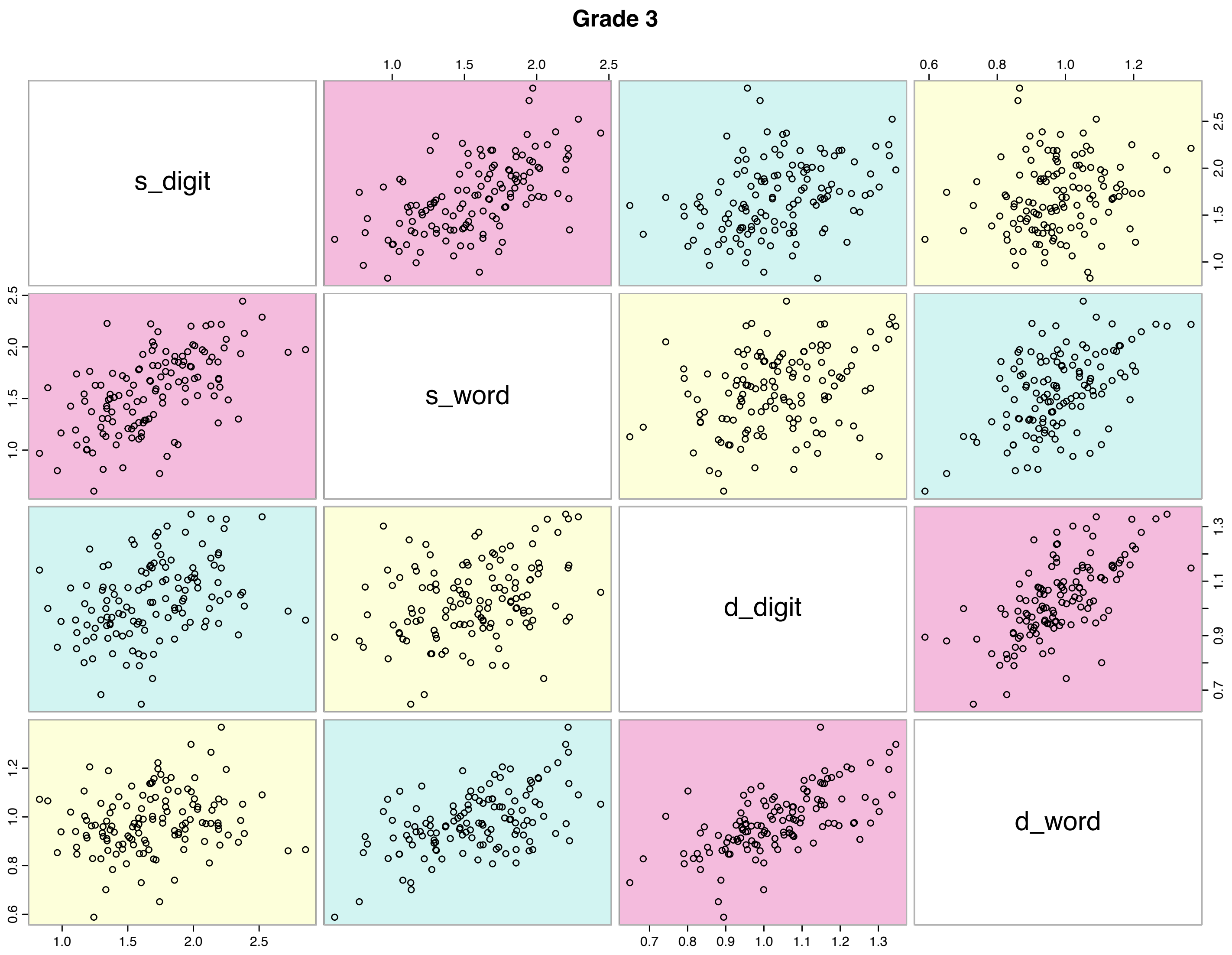

Supplementary Figure 2. Bivariate scatterplots among serial and discrete digits and words in Grade 3. Darker colors indicate stronger correlations. (All data points in items per second).

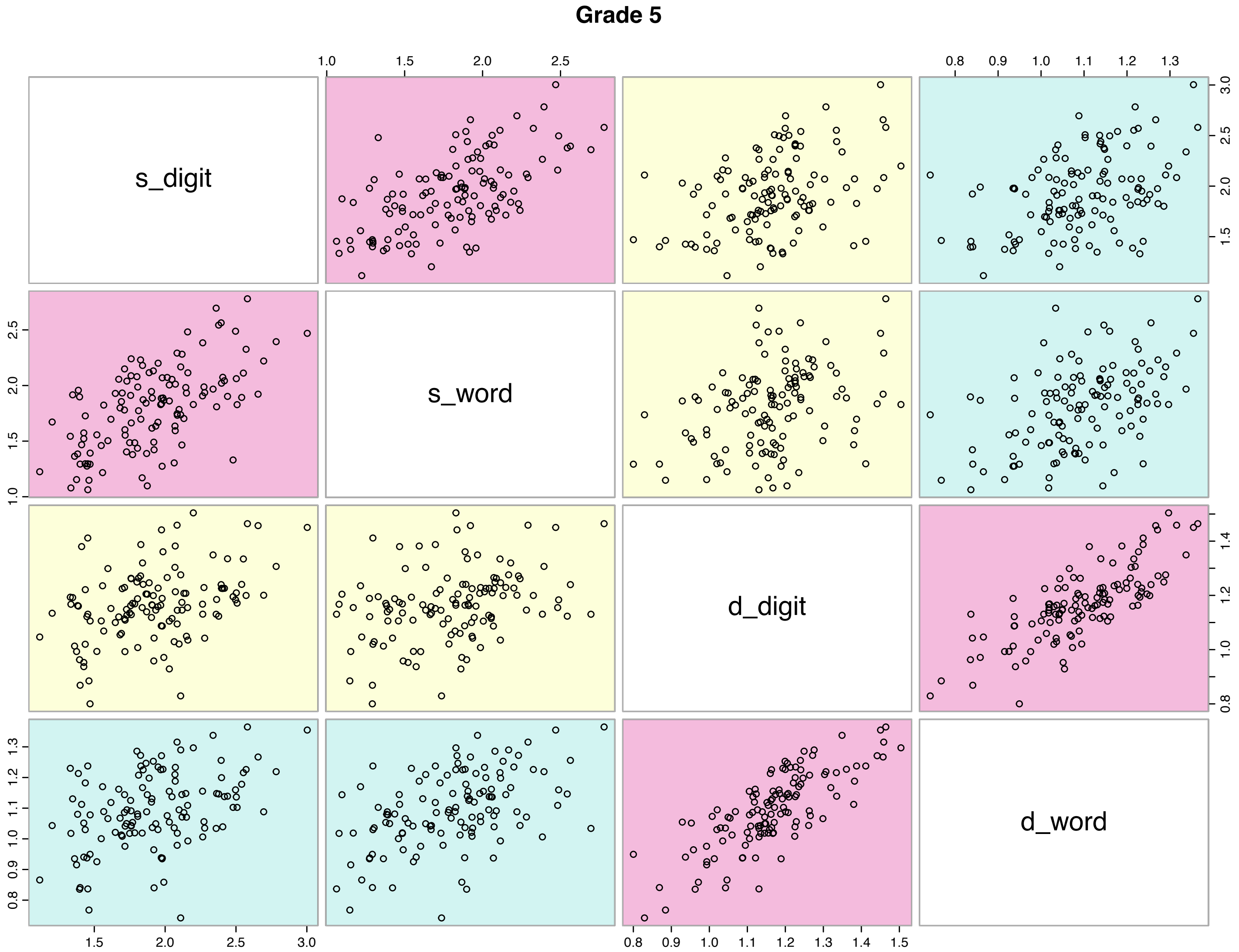

Supplementary Figure 3. Bivariate scatterplots among serial and discrete digits and words in Grade 5. Darker colors indicate stronger correlations. (All data points in items per second).
